# Practices and Challenges of Household Solid Waste Management in Woldia Town, Northeastern Ethiopia

**DOI:** 10.5696/2156-9614-11.30.210605

**Published:** 2021-05-28

**Authors:** Silamlak Birhanu Abegaz, Kindu Alem Molla, Seid Ebrahim Ali

**Affiliations:** Woldia University, Faculty of Natural and Computational Sciences, Department of Biology

**Keywords:** household, solid waste management practices, Woldia town Ethiopia

## Abstract

**Background.:**

The rapid growth of urban populations has led to a dramatic increase in urban waste generation with environmental and public health problems associated with water contamination, emission of toxic and noxious fumes, soil contamination and introduction of disease vector populations.

**Objectives.:**

The aim of the present study was to assess solid waste disposal practices and challenges of solid waste management in Woldia town, northeastern Ethiopia.

**Methods.:**

A descriptive research design was employed, using a survey method and naturalistic observational methods. A total of 236 households were surveyed, including waste collectors and cleaners, waste pickers, and 20 students and ten teachers from Woldia secondary and preparatory schools. A purposive sampling technique (made up of municipal officers, scavengers (waste pickers), waste collectors and cleaners and a simple random sampling technique (of teachers, students and *kebeles* of the study area) was employed, then proportional allocation was made for each randomly selected *kebeles* based on population size to determine the sample. Finally, survey questionnaire, interview, focus group discussion and observation were used as data collecting tools. Bivariate and multivariate analyses were also employed.

**Results.:**

The current study revealed that educational status (AOR = 10.92, 95% CI = (3.12–38.27)), occupational status (AOR = 8.08, 95% CI = (2.08–31.31)), monthly income (AOR = 5.72, 95% CI = (1.55–21.13)), and age (AOR = 2.53, 95% CI = (1.04–6.19)) were found to be the major factors associated with solid waste management practices. Additionally, shortage of storage materials, lack of alternative waste disposal options, household attitudes, and lack of awareness were the major challenges for low performance of solid waste management practices of the local government and households. The focus group individuals and interviewed participants indicated that solid waste management practices were poor.

**Conclusions.:**

The present study revealed that solid waste management practices in Woldia town remain inadequate. Therefore, efforts by the municipality and other stakeholders are needed to mitigate the problem of waste management and disposal practices.

**Participant Consent.:**

Obtained

**Ethics Approval.:**

The study was approved by the Institutional Research Ethics Review committee (IRERC) of Woldia University

**Competing Interests.:**

The authors declare no competing financial interests.

## Introduction

The unrestricted production of solid waste and its unsafe disposal is becoming a global problem.^1^ Urbanization, population growth and modernization have rapidly increased the rate of municipal solid waste (MSW) production and disposal in many cities around the globe.^2^,^3^ Municipal solid waste consists of everyday items such as product packaging, yard trimmings, furniture, clothing, bottles and cans, food, newspapers, appliances, electronics, and batteries. Sources of MSW include residential waste (including waste from multi-family housing) and waste from commercial and institutional locations, such as businesses, schools, and hospitals.^4^ Similarly, local residents and municipal authorities defined solid waste as material which no longer has any value to its original owner, which is then discarded in town, including kitchen waste and garden trimmings, paper, glass, metals and plastics, ash, dust and street sweepings. According to United States Environmental Protection Agency (USEPA) definitions,^4^ MSW does not include industrial, hazardous or construction and demolition waste. Once generated, MSW must be collected and managed. However, solid waste management (SWM) has remained a challenge to both developed and developing countries globally. As a low-and middle-income country (LMIC), Ethiopian cities and towns also deal with the environmental costs of pollution due to unregulated landfill waste. The rapid increase in the volume of waste is one aspect of the environmental crisis in Woldia town. As much of the waste is landfilled, that represents a loss of materials that could be reused, recycled, or converted to energy to displace the use of virgin materials. The primary SWM activities involve the collection, storage, transportation, processing, treatment, recycling, and final disposal of waste.^1^ In urban cities of LMICs, management of solid waste remains poor.^2^,^3^ Good solid waste management practices protect the environment from the risk of ineffective disposal system of solid wastes. Discarded solid wastes generate a large volume of polluted leachates that contains high concentrations of toxic compounds which pose a risk to the ecosystem^5^ and may introduce disease vector populations.^4^ Improper waste disposal and management may result in health and environmental problems and may become life threatening. Water, soil, and air pollution have been attributed to improper disposal and management of solid waste.^3^ The rate of solid waste production is increasing with population growth, technological development, and changes in lifestyle.^6^ In LMICs, improper handling and disposal of solid waste contributes to high levels of mortality and morbidity.^7^ A study conducted by Selin reported that discarded solid wastes can have severe health effects on residents due to its infectious, toxic or radioactive nature.^8^ According to Kimani, in conjunction with the United Nations Environmental Programme (UNEP) the public health effects from municipal wastes include severe illness among residents, including skin disorders, respiratory abnormalities, abdominal and intestinal problems, dental disorders, ear infections, skeletal muscular systems (back pain), central nervous system (neurological impairment), eye infections, blood disorders (anemia), malaria, chicken pox, septic wounds and congenital abnormalities, cardiovascular disease and lung cancer.^9^

**Abbreviations**
*LMIC*: Low- and middle-income country
*MSSE*: Micro- and small-scale enterprise
*MSW*: Municipal solid waste
*SWM*: Solid waste management

The present study is also relevant to the development of pollution control technologies, as more rigorous legislation and strategies on waste handling and disposal are needed to minimize environmental pollution and risks to public health. Most LMIC countries recognize that solid waste management is needed to secure the safety of the environment and human health.^10^ However, SWM is a critical challenge for LMIC countries including Ethiopia because of the social, economic, and environmental implications. Ethiopia is facing rapid urbanization leading to overcrowding and development of informal settlements with poor waste management practices. Solid waste management is becoming a major public health and environmental concern in urban areas of Ethiopia, as only 2% of the population receives solid waste collection services.^11^ Ethiopia has ratified several international conventions that have important implications for solid waste management in the country.^10^

Woldia is a rapidly growing town in Ethiopia. A study done by Semaw indicated that solid waste management in Woldia town is inadequate primarily due the absence of suitable waste disposal sites and suggests that the selection of suitable waste disposal sites may address this problem.^12^

However, that study was not able to examine detailed aspects of solid waste management practices and challenges. Solid waste generation rates in Woldia town are increasing along with population growth rates over time. This rapid increase in population together with rapid development in the town has produced increasing volumes of solid waste, and consequently greater public health risks. The objectives of the present study were to assess solid waste disposal practices and challenges of solid waste management in Woldia town, Ethiopia, to examine the institutional and community participation in solid waste management practices, and to identify the types of solid wastes from different sites in the town.

## Methods

**Figure 1:**
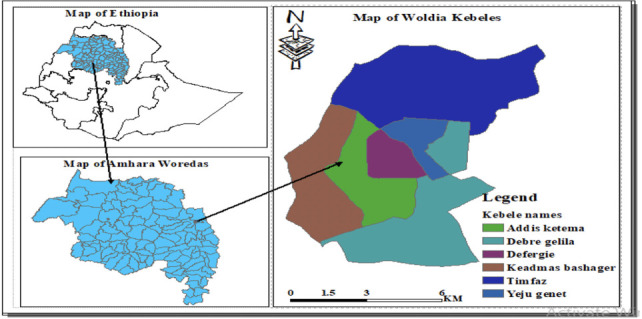
Map of the study area

Woldia town *(Figure 1)* is located in the North Wollo Zone, Amhara Regional State of Ethiopia, at 11°50' N and 39°36' E. The town is located north of Addis Ababa and bordered by Habru Woreda in the south, Gubalfto Woreda in the west and east, and Raya Kobo Woreda in the north and northeast. Woldia town, the capital of the North Wollo Zone, is situated 521 km from the capital city of Addis Ababa, 370 km from Bahir Dar, 120 km from Dessie, and 260 km from Mekele. Based on the estimation of the Central Statistical Agency of Ethiopia, 2007, the total population of the town is 72 294 and total number of households is 14 466.^13^

### Study design

The present study employed a descriptive research design method using survey and observational methods.

#### Sample size determination

To select households to assess the current status of household solid waste management practices in Woldia town, the sample size was determined using the statistical method of Cochran *(Equation 1),* assuming a non-response rate of 5% and design effect of 1.5. 14

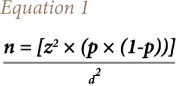



Thus,

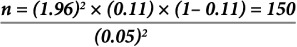
Where n= sample size of housing units, z = standardized normal variable and its value corresponding to a 95% confidence interval equals 1.96, d = allowable error (0.05), and P= 0.11 (11%) for the proportion of households expected to practice proper household solid waste management.^15^

Adding 5% for nonresponses and multiplying the sample size by 1.5 for design effect, the sample size was increased to 236 respondents (households).^15^ All waste collectors and cleaners in the town (60), all officers accountable for Woldia town sanitation (11), 236 households, 43 scavengers (waste pickers), and 20 students (10 from Woldia Millennium secondary school and 10 from Woldia secondary and preparatory school) and 10 teachers (5 from Woldia Millennium secondary school and five (5) from Woldia secondary and preparatory school) were also considered due to assumptions that they would provide better information on the topic and due to proximity to the town.

### Sampling

**Figure 2:**
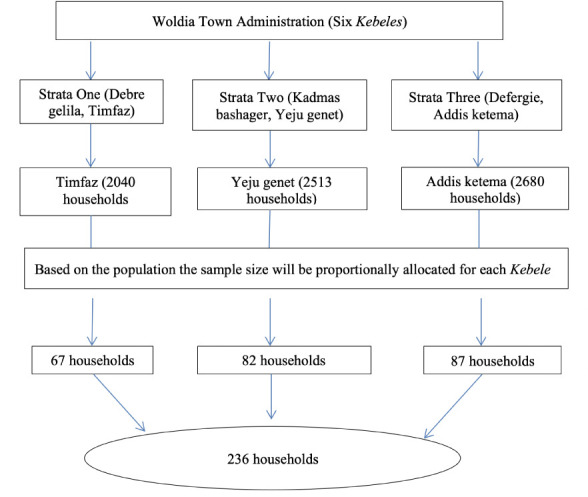
Schematic representation of sampling procedure

The purposive sampling technique was used to select municipal officers, scavengers (waste pickers), all of the waste collectors and cleaners in Woldia town and the simple random sampling technique was also used to select students and teachers from secondary and preparatory schools. For household selection, based on geographical location of the town, the six *kebeles* were divided into three strata. A *kebele* is the smallest administrative unit in Ethiopia, similar to a ward or a neighborhood. Next, one *kebele* was selected from each stratum using the random sampling technique. Finally, households were selected proportionally from the three *kebeles* based on their population size *(Figure 2).*

### Data collection instruments

Structured and standardized questionnaires were adapted from the Federal Democratic Republic of Ethiopia Demographic and Health Survey (EDHS), English version *(Supplemental Material 1).*^16^ The questionnaire was further developed by reviewing the literature and relevant factors such as financial budget and logistics, provision of municipal infrastructure, and the perceptions and waste disposal practices of communities were taken into consideration.^16^ The statements in the questionnaire assessed household, solid waste picker, and cleaner understanding of the practice and challenges of solid waste management. Questions were translated into the local language (Amharic) by language experts. The questionnaire included both close-ended and open-ended questions, demographic and socio-economic characteristics, and the current situation of household solid waste management practices. For household respondents, those who were above 18 years old and had resided in the town for two or more years were eligible for the questionnaire survey. In addition, the head of household was the questionnaire respondent. Illiterate respondents were assisted by questionnaire administrators.

### Interview process

For the qualitative study, a total of 114 participants consisting of officers (11) from the town municipality sanitation office, scavengers (waste pickers) (43), and waste collectors and cleaners (60) were purposefully selected as key informants. Interview guides were adapted from Selin,^8^ and can be found in Supplemental Material 2.

#### Observation

Using a checklist, photos were taken of the area for visualization *(Supplemental Material 3 and 4)*. Household level practices were observed, including the handling and collection of SWs, transport and disposal methods and disposal site, waste management practices along the road, drainage canals, municipality dustbins, open spaces, business areas, and marketplace.

#### Focus group discussion

Focus group discussion was also used to collect data from a total of 30 teachers and students. Students and teachers from both schools (Woldia Millennium secondary school and Woldia secondary and preparatory school) were grouped into three focus group discussions, each containing 8–13 individuals, and conducted in the two secondary and preparatory schools of Woldia town. Focus group participants were a small, carefully selected group and homogenous in social composition. The town sanitation officers and *kebele* administrators helped in identifying focus group participants in order to include participants from different religions, age, gender, classes and educational levels. Issues related to practices and challenges of household solid waste management were discussed in each focus group session.

### Study variables

Household solid waste management practices across Woldia Town and its challenges were regarded as dependent variables. Gender, age, family size, educational status, income and years of residency were considered as demographic independent variables. Awareness of solid waste management, willingness to pay for waste collection services, distance from the main road, attitude, distance from waste containers and availability of other disposal places were considered as socio-economic factors as well as institutional factors like access to waste collection.

### Data analysis

Quantitative data were analyzed by means of descriptive statistics and qualitative data were analyzed by narration. Data were first checked manually for completeness and then coded, entered, and analyzed using Statistical Package for the Social Sciences (SPSS) version 20 software using descriptive statistics such as frequency and percentage. Finally, bivariate and multivariate analysis were performed to identify significant factors associated with household solid waste management practices.

### Ethics approval and consent

The study was approved by the Institutional Research Ethics Review committee (IRERC) of Woldia University after a review of the research protocol. The respondents were informed about the purpose of the study, and their oral consent was obtained. The respondents' right to refuse or withdraw from participating was fully maintained and the information provided by each respondent was kept strictly confidential. Before entering the study area to collect data, local authorities and community leaders were given information about the study objective.

## Results

A total of 236 households participated in the questionnaire survey. Out of the total study subjects, 117 (49.6%) were male and 119 (50.4%) were female. The largest group included those 41–60 years of age (44.9%). Furthermore, 69 (29.2%) and 42 (17.8%) respondents had completed secondary school and diploma level education, respectively. One hundred and fifteen (115, 48.7%) of the participants were married *(Table 1).* Concerning occupation, 189 (36.9%) were merchants and 139 (27.1%) of respondents were government employees. Regarding monthly income, most of the respondents (63, 26.7%) earned <1000 Ethiopian Birr (roughly 24.75 USD).

**Table 1:** Socio-demographic Characteristics

**Variables**	**Category**	**Frequency**	**%**
Gender	Male	117	49.6
	Female	119	50.4
	**Total**	236	100.0
Age	18–40	69	29.2
	41–60	106	44.9
	>60	61	25.8
	**Total**	236	100.0
Educational status	No formal education and illiterate	33	14.0
	No formal education but literate	38	16.1
	Primary	54	22.9
	Secondary	69	29.2
	Diploma and above	42	17.8
	**Total**	236	100.0
Marital status	Single	22	9.3
	Married	115	48.7
	Divorced	54	22.9
	Widowed	45	19.1
	**Total**	236	100.0
Occupational status	Employer	37	15.7
	Daily worker	64	27.1
	Merchant	78	33.1
	Others*	57	24.2
	**Total**	236	100.0
Monthly household income (in Birr)*	<1000	63	26.7
	1000–2500	89	37.7
	2501–3500	44	18.6
	>3500	40	16.9
	**Total**	236	100.0
Family size	1–3	86	36.4
	4–6	128	54.2
	>6	22	9.3
	**Total**	236	100.0
Years of stay in Woldia town	2 years	149	63.1
	> 2 years	87	36.9
	**Total**	236	100.0
Housing condition	Private rental	100	42.4
	*Kebele* rental	86	36.4
	Private house	50	21.2
	**Total**	236	100.0

^*^ <1000 Birr = <24.75 USD, 1000–2500 Birr = 24.75–61.85 USD, 2501–3500 Birr = 61.85–86.55 USD

Table 2 shows 153 (64.8%) households do not collect or store solid wastes separately based on type. Plastics and wastes other than ash, food and “khat” or “chat” leftovers are separated by 58 (24.6%) of households. Chat is a flowering plant traditionally consumed in Ethiopia as a stimulant. The remaining leaves, twigs, and stems (leftovers) thrown away to decompose or be scavenged by livestock, especially goats, with considerable waste.^17^ Most of the respondents (125, 53.0%) indicated that waste-related laws and regulations were posted in the town. However, 139 (58.9%) respondents reported that the laws and regulations were not implemented by the municipal authorities. In the present study, 74 (31.4%) of the respondents lacked knowledge of the need for separation of solid wastes.

**Table 2:** Households and Municipal Authority Solid Waste Management Practices

**Variables**	**Category**	**Frequency**	**%**
Do households collect and store solid wastes separately based on type	Yes	83	35.2
	No	153	64.8
	**Total**	236	100.0
Types of household wastes separated by households	Plastic	58	24.6
	Ash/solid remains of fire	28	11.9
	Food leftovers	50	21.2
	Chat leftovers	42	17.8
	Others	58	24.6
	**Total**	236	100.0
Reasons for not separating solid waste	Lack of knowledge	74	31.4
	Not responsible	71	30.1
	Not important	58	24.6
	Don’t know	33	14.0
	**Total**	236	100.0
Are waste-related laws and regulations posted in town	Yes	125	53.0
	No	111	47.0
	**Total**	236	100.0
Are waste-related laws and regulations implemented by municipal authorities	Not at all	139	58.9
	Weak regulation	72	30.5
	Strong regulation	25	10.6
	**Total**	236	100.0

### Factors associated with solid waste management practices during bivariate and multivariate analysis among households in Woldia town

The binary logistic regression analysis results in Table 3 showed that average age of households (41–60), lower educational status, marital status of married or widowed, occupational status, monthly income of 2501–3500 birr (61.85–86.55 USD), and family size of 1–3 and 4–6 were found to be significantly associated with poor waste management practices.

**Table 3:** Factors Associated with Solid Waste Management Practices among Households in Woldia Town

**Variables**	**Waste management practice**		**OR (95%CI)**			

	Good n (%)	Poor n (%)	COR (95% CI)	p- value	AOR (95%CI)	p-value
*Gender*						
Male	43 (18.2)	74 (31.4)	1		1	
Female	46 (19.5)	73 (30.9)	0.922 (0.545–1.561)	.763	1.30 (0.63–2.69)	.486
*Age*						
18–40	22 (9.3)	47 (19.9)	2.36 (1.16–4.81)	.018*	2.44 (0.92–6.48)	.073
41–60	35 (14.8)	71 (30.1)	2.24 (1.17–4.27)	.014*	2.53 (1.04–6.19)	.041*
>60	32 (13.6)	29 (12.3)	1		1	
*Educational status*						
No formal education and illiterate	7 (3.0)	26 (11.0)	3.71 (1.33–10.41)	.013*	8.74 (2.31–33.16)	.001*
No formal education but literate	8 (3.4)	30 (12.7)	3.75 (1.40–10.06)	.009*	4.45 (1.33–14.86)	.015*
Primary	10 (4.2)	44 (18.6)	4.40 (1.76–10.99)	.002*	10.92 (3.12–38.27)	.000*
Secondary	43 (18.2)	26 (11.0)	0.61 (0.28–1.31)	.204	0.71 (0.25–2.01)	.521
Diploma and above	21 (8.9)	21 (8.9)	1		1	
*Marital status*						
Single	1 (0.4)	21 (8.9)	1		1	
Married	48 (20.3)	67 (28.4)	0.07 (0.01–0.51)	.009*	0.08 (0.01–0.73)	.026*
Divorced	19 (8.1)	35 (14.8)	0.09 (0.01–0.70)	.022*	0.21 (0.02–2.08)	.182
Widowed	21 (8.9)	24 (10.2)	0.05 (0.01–0.44)	.006*	0.07 (0.01–0.74)	.027*
*Occupational status*						
Employer	13 (5.5)	24 (10.2)	1		1	
Daily worker	27 (11.4)	37 (15.7)	0.74 (0.32–1.72)	.486	1.64 (0.43–6.19)	.469
Merchant	32 (13.6)	46 (19.5)	0.78 (0.35–1.75)	.546	3.06 (0.84–11.13)	.090
Others	17 (7.2)	40 (16.9)	1.28 (0.53–3.08)	.590	8.08 (2.08–31.31)	.003*
*Monthly income*						
<1000 Birr*	24 (10.2)	39 (16.5)	1		1	
1000–2500	41 (17.4)	48 (20.3)	0.72 (0.37–1.39)	.328	0.72 (0.29–1.78)	.477
2501–3500	9 (3.8)	35 (14.8)	2.39 (0.98–5.84)	.055	5.72 (1.55–21.13)	.009*
>3500	15 (6.4)	25 (10.6)	1.03 (0.45–2.32)	.952	0.67 (0.13–3.46)	.633
*Family size*						
1–3	30 (12.7)	56 (23.7)	0.19 (0.04–0.85)	.030*	0.10 (0.01–0.83)	.033*
4–6	57 (24.2)	71 (30.1)	0.13 (0.03–0.56)	.006*	0.06 (0.01–0.42)	.005*
>6	2 (0.8)	20 (8.5)	1		1	
*Year of stay in Woldia town*						
2 years	56 (23.7)	93 (39.4)	1.02 (0.59–1.75)	.958	1.25 (0.59–2.67)	.561
> 2 years	33 (14.0)	54 (22.9)	1		1	
*Housing condition*						
Private rental	40 (16.9)	60 (25.4)	0.47 (0.22–1.02)	.055	0.45 (0.15–1.37)	.160
*Kebele* rental	37 (15.7)	49 (20.8)	0.42 (0.19–0.91)	.028*	0.37 (0.11–1.21)	.099
Private house	12 (5.1)	38 (16.1)	1		1	

*<1000 Birr = <24.75USD, 1000–2500 Birr = 24.75–61.85 USD, 2501–3500 Birr = 61.85–86.55 USD

* Statistically significant at p<0.05, OR=odds ratio, AOR=adjusted odds ratio, COR=crude odds ratio, CI=confidence interval

Table 4 shows the major challenges to waste management practices in Woldia town. A total of 120 (50.8%) households reported a shortage of waste disposal materials, particularly trash disposal bags. Similarly, most of the respondents reported a lack of alternative waste disposal sites, inadequate access in residential areas, inadequate access to micro- and small-scale enterprise (MSSE) services, household's lack of positive attitudes towards SWM, household's lack of awareness of solid waste management practices (92 (39.0%)), unwillingness to pay for waste collection services (125 (53.0%)), lack of space in backyard for waste disposal (131 (55.5%)), and distance of houses from the main road.

**Table 4:** Major Challenges in Household Solid Waste Management

**Variable**	**Category**	**Frequency**	%
Which trash disposal equipment(s) are/is limited?	Skip bin	57	24.2
	Trash disposal bags	120	50.8
	Plastic bins	59	25.0
	**Total**	236	100.0
Alternative waste disposal options	Piece of land with no buildings and accessible to public	95	40.3
	Green waste landfills	47	19.9
	Municipal solid waste landfills	61	25.8
	Managed landfills	33	14.0
	**Total**	236	100.0
Is there adequate access to the residential area?	Yes	77	32.6
	No	159	67.4
	**Total**	236	100.0
Access to micro- and small-scale enterprises services	Adequate	43	18.2
	Inadequate	132	55.9
	None	61	25.8
	**Total**	236	100.0
Do households have positive attitude towards SWM?	Yes	86	36.4
	No	150	63.6
	**Total**	236	100.0
Awareness of households on SWM	Inadequate	92	39.0
	Moderate	85	36.0
	Adequate	59	25.0
	**Total**	236	100.0
Willing to pay for waste collection services	Yes	111	47.0
	No	125	53.0
	**Total**	236	100.0
Is there space in backyard for waste disposal?	Yes	105	44.5
	No	131	55.5
	**Total**	236	100.0
Distance of houses from main road (meters)	<51	73	30.9
	51–100	77	32.6
	101–150	48	20.3
	> 150	38	16.1
	**Total**	236	100.0

## Discussion

The present study was carried out to assess the challenges and the household solid waste management practices of the municipality and communities in Woldia town. Descriptive data analysis was performed to summarize the sociodemographic status of the respondents. In the present study, gender of respondents and duration of residency in Woldia town did not have any relationship with solid waste management practices. However, a comparable study performed in Dire Dawa indicated that respondents who lived there less than one year were 0.5 times less likely to have improper waste management practices compared to those who had lived there a year or more.^18^ Another study in Ambo town revealed an insignificant relationship between length of residency and household solid waste management practices.^16^ The present study also revealed that educational status was associated with household waste management practices. A similar study in Dire Dawa showed that those who were illiterate were two times more likely to have improper waste management practices compared to those who were literate.^18^ From a housing perspective, residing in private or rental houses showed no association with solid waste management practices. However, a study conducted by Haile in Ambo town reported that of 200 households, 55% of the respondents live in rental houses either privately or *kebele*, and the respondents were aware of solid waste management practices.^16^

The current study found that plastics and other wastes (kitchen waste, glass, and dust) were the prominent municipal wastes generated by households. However, a study conducted in Dire Dawa by Daniel *et al.* indicated that plastic, ash and “Khat” or “Chat” leftovers constitute the major bulk of the waste generated by households.^18^ In the present study, the community and municipality did not collect or store solid wastes separately based on type *(Table 2).* Hence, most were unaware of the proper management of solid wastes. On the contrary, a study in Kenya found that 94.2% of respondents were aware of hazards brought about by incorrect solid waste management practices, yet only 26.2% practiced proper waste management.^19^ In the present study, most of the participants also indicated that laws and regulations related to solid waste management practices are posted, but there is little implementation of these laws and regulations. A similar study done by Daniel *et al.* found that when the law enforcement and regulations are weak, improper waste management practices are 2.8 times more likely than when waste laws and regulations are enforced.^18^

Woldia is a rapidly growing town in Ethiopia. As a result, solid waste management is becoming a major public problem and environmental concern in *kebeles* across the town. The present study found that average age of households between 41–60 years, educational status of no formal education and illiterate, no formal education but literate, or primary education only, and marital status of married or widowed were found to be significant factors associated with poor waste management practices. Similarly, the study carried out in Dire Dawa by Daniel *et al.* reported that 352 (69%) of respondents disposed of solid wastes improperly.^18^ According to the report, the educational level of the households and marital status of the respondents were associated with household solid waste management in the study area. In the current study, findings related to factors like occupational status; jobs, monthly income, and family size were not in agreement with the study done by Daniel *et al.*^18^ However, awareness of households on solid waste management, attitude of households on solid waste management, distance of the house from the main road were also additional factors considered in the study conducted by Daniel *et al.*^18^ In the current study, shortage of waste disposal materials, lack of alternative waste disposal options, inadequate access to residential areas, access to MSSE services, household attitudes on SWM, lack of awareness of households on SWM, unwillingness to pay for waste collection services, limited availability of space in the backyard for waste disposal and distance of houses from the main road were the major challenges related to poor solid waste management practices for households and municipal authorities. However, degrees of influence varied across *kebeles (Table 4).* A study conducted by Daniel *et al.* revealed that law enforcement and access to micro- and small-scale enterprises were the major constraints for waste management practices by the local authorities and households.^18^

Waste management is a growing problem in Ethiopia.^20^ In many cities of the country, waste management is poor and solid wastes are dumped along roadsides and into open areas, endangering human health and attracting vermin. Only 12% (4% rural and 8% urban) of the population uses improved sanitation facilities.^21^

According to United Nations Economic and Social Council, population growth and the rate of urbanization are rapidly increasing throughout the African continent.^22^ However, the technology, technical knowledge, financial resources, culture, attitude, and understanding required to properly manage solid wastes remain inadequate. Urbanization along with inadequate waste management practices such as widespread disposal of waste in water bodies and dumping alongside the road and in uncontrolled dump sites aggravates the problems of generally low sanitation levels across the African continent. Studies conducted in major towns and cities of Ethiopia have indicated that solid wastes are not appropriately handled and managed, mainly due to institutional, regulatory, financial, technical, and public participation problems, similar to the focus group and interview findings in the present study.^23^, ^24^

## Conclusions

The current study revealed that the solid waste disposal practices of most households were poor and unregulated. Educational and marital status, family size, average monthly income, occupation and age were found to be associated with solid waste management practices. Additionally, shortage of storage materials, lack of alternative waste disposal options, inadequate access in residential areas, lack of access to MSSE services, household attitude on SWM, lack of household awareness of SWM, willingness to pay for waste collection services, availability of space in the backyard for waste disposal, and distance of household from the main road were the major challenges for low performance of solid waste management practices by municipal authorities and households. The focus group and interviewed officers, scavengers, and waste collectors and cleaners indicated that solid waste management practices were poor. They also confirmed that institutional, financial, logistical, and public participation problems were the major barriers to effective solid waste management practices. Further efforts are needed to mitigate the problem of inadequate waste management practices in Woldia town by creating awareness among communities and stakeholders.
