# Supplementary material for: Practices and Challenges of Household Solid Waste Management in Woldia Town, Northeastern Ethiopia
Source: J Health Pollut. 2021 May 28;11(30):210605. doi: 10.5696/2156-9614-11.30.210605 (PMC8276726; doi:10.5696/2156-9614-11.30.210605)
Supplement: Supplementary file 1 [file Abegaz_Supplemental_Material_1.docx]

**Supplemental Material 1**

**Questionnaires for household data collection**

**WOLDIA UNIVERSITY
Faculty of Natural and Computational Science
Department of Biology**

The purpose of this questionnaire is to gather information/data for research conducted under the title of **PRACTICES AND CHALLENGES OF HOUSEHOLD SOLID WASTE MANAGEMENT IN WOLDIA TOWN, NORTHEASTERN ETHIOPIA.** This questionnaire consists of questions that focus on the demographic characteristics of respondents and the practices and challenges of household solid waste management by the municipal authority and community in Woldia town. The success of this study depends on the quality and trustworthiness of the responses given by the research participants (respondents). You are kindly requested to answer the questions carefully for the successful completion of this research. Finally, we would like to express our appreciation for your willingness to be part of this research project.

Thank you!

**Part one: Demographic characteristics**

**Give answer for the following questions by using a tick mark “**✓**“in the box of your choice**

| **Gender** | Male |
| --- | --- |
|  | Female |
| **Age** | 18-40  41-60  >60 |
| **Educational status** | Illiterate  Literate  1-8  9-10  11-12  College/university |
| **Marital status** | Single  Married  Divorced  Widowed |
| **Occupational status** | Government employee  Daily worker  Farmer  Others |
| **Monthly household income (in Birr)** | <1000  1000-2500  2501-3500 |
| **Family size** | 1-3  4-6  > 7 |
| **Years of stay in Woldia town** | 2 years  > 2 years |
| **Housing conditions** | Private rental  *Kebele* rental  Private house |

**Part two: Households and municipal authority solid waste management practices**

| **Do the households collect and stored solid wastes separately based on type?** | Yes  No |
| --- | --- |
| **Types of household wastes separated by households** | Plastics  Ash/solid remains of a fire  Food leftovers  “Khat” or “Chat” leftovers  Others |
| **Reasons for not separating solid waste** | Lack of knowledge  Not responsible  Not important  I don’t know |
| **Are waste related laws and regulations posted in town?** | Yes  No |
| **Are waste related laws and regulations implemented by the municipality?** | Not at all  Weak regulation  Strong regulation |

**Part three: Challenges in municipal authorities and households for solid waste management**

| **Which trash disposal equipment(s) are/is limited?** | Skip bin  Rash disposal bags  Plastic bins |
| --- | --- |
| **Alternative waste disposal options** | Piece of land with no buildings and accessible to public  Hole/hollow places  Municipal solid waste landfills  Managed landfills |
| **Access to residential area** | Yes  No |
| **Access to micro- and small-scale enterprise service** | Adequate access  Inadequate  None |
| **Do households have positive attitude towards solid waste management?** | Yes  No |
| **Awareness of households on solid waste management** | Inadequate  Moderate    More adequate |
| **Willing to pay for waste collection services?** | Yes  No |
| **Availability of space in backyard for waste disposal?** | Yes  No |
| **Distance of houses from main road (meters)** | < 50  51-100  101-150  >150 |

**Part four:** **Open-ended questions for respondents**

How and where do you dispose wastes generated in your home?_

_______________________________________________________________________

Do you know about solid waste? ________________________________________________________________________

Did you know how solid waste is managed? ________________________________________________________________________

Does the municipal authority allocate a sufficient budget for solid waste management in the town?

If not why? __________________________________________________________________

Do you get training on solid waste management by the municipal authority?

If not, why?

______________________________________________________________________

| What materials do you use for storage of wastes produced in your home?  _____________________________________________________________________________  **Source:** Adapted from Ethiopia demographic health survey report.**^16^** |
| --- |
