# Supplementary material for: Practices and Challenges of Household Solid Waste Management in Woldia Town, Northeastern Ethiopia
Source: J Health Pollut. 2021 May 28;11(30):210605. doi: 10.5696/2156-9614-11.30.210605 (PMC8276726; doi:10.5696/2156-9614-11.30.210605)
Supplement: Supplementary file 2 [file Abegaz_Supplemental_Material_2.docx]

**Supplemental Material 2**

**Interview guide**

***Opening questions***

How long have you lived in Woldia town? What are your general thoughts on living here?

***Awareness on waste management and risk for health effects***

1. What does garbage/waste mean to you? If I say the word waste management, what do you think about? What are your opinions about taking care of garbage (e.g. waste management)? Do you believe garbage can be dangerous and if so, in what way and to whom?
2. Do you look differently on different types of waste and in what way? Do you know which type of waste classifies as hazardous waste?
3. Are you aware of some health effects that can be caused by improper disposal of waste, e.g., through burning and by waste left in the environment?

***Personal possibilities (knowledge, time and money)***

1. Do you have an education and if so, what is your education level? Has it included any environmental topics mentioning waste management?

***Personal habits (routines in daily life)***

1. During everyday life do you feel that it would be possible to collect and sort waste? What would be a motivating factor for you?

***Surroundings (nearby access)***

1. Where can you dispose of your waste? Can you describe how you get rid of waste? Are there any trash collecting cans or other solutions for handling waste?

***Attitudes (values and opinions)***

1. What are your personal thoughts on collecting, sorting and recycling of waste? Do you think that it is important to take care of garbage in a wider perspective (for future generations, nature and humans)?

| Source: Adapted from **Selin E.** Solid Waste Management and Health Effects: A Qualitative Study on Awareness of Risks and Environmentally Significant Behaviour. Biotech Thesis submitted to UMEA University, Mutomo Kenya; Feb 22, 2013 |
| --- |
