# Supplementary material for: Practices and Challenges of Household Solid Waste Management in Woldia Town, Northeastern Ethiopia
Source: J Health Pollut. 2021 May 28;11(30):210605. doi: 10.5696/2156-9614-11.30.210605 (PMC8276726; doi:10.5696/2156-9614-11.30.210605)
Supplement: Supplementary file 3 [file Abegaz_Supplemental_Material_3.docx]

**Supplemental Material 3**

**Observation checklist**

Does the municipality provide temporary storage in the town?

Is the stakeholder involved in waste collection?

Does the municipality have good infrastructure for solid waste management, such as vehicles?

Does the municipality have a good disposal site?

Does municipality have a good collection site?

Is there any participation by the community in waste management practices?

Does the town practice the 3R’s (Reduce, Reuse and Recycle)?

Are all family members responsible for waste collection and disposal in multi-family housing?

What are the different materials dumped in the community’s dwellings?

Source: Adapted from **Haile A.** Determinants of Effective Household Solid Waste Management Practices: the Case of Ambo Town – West Showa Zone, Thesis submitted to Mekelle University, Ethiopia; 2011.
