# Supplementary material for: Practices and Challenges of Household Solid Waste Management in Woldia Town, Northeastern Ethiopia
Source: J Health Pollut. 2021 May 28;11(30):210605. doi: 10.5696/2156-9614-11.30.210605 (PMC8276726; doi:10.5696/2156-9614-11.30.210605)
Supplement: Supplementary file 4 [file Abegaz_Supplemental_Material_4.docx]

**Supplemental Material 4

Photo of solid wastes dumped in open spaces in Woldia town**


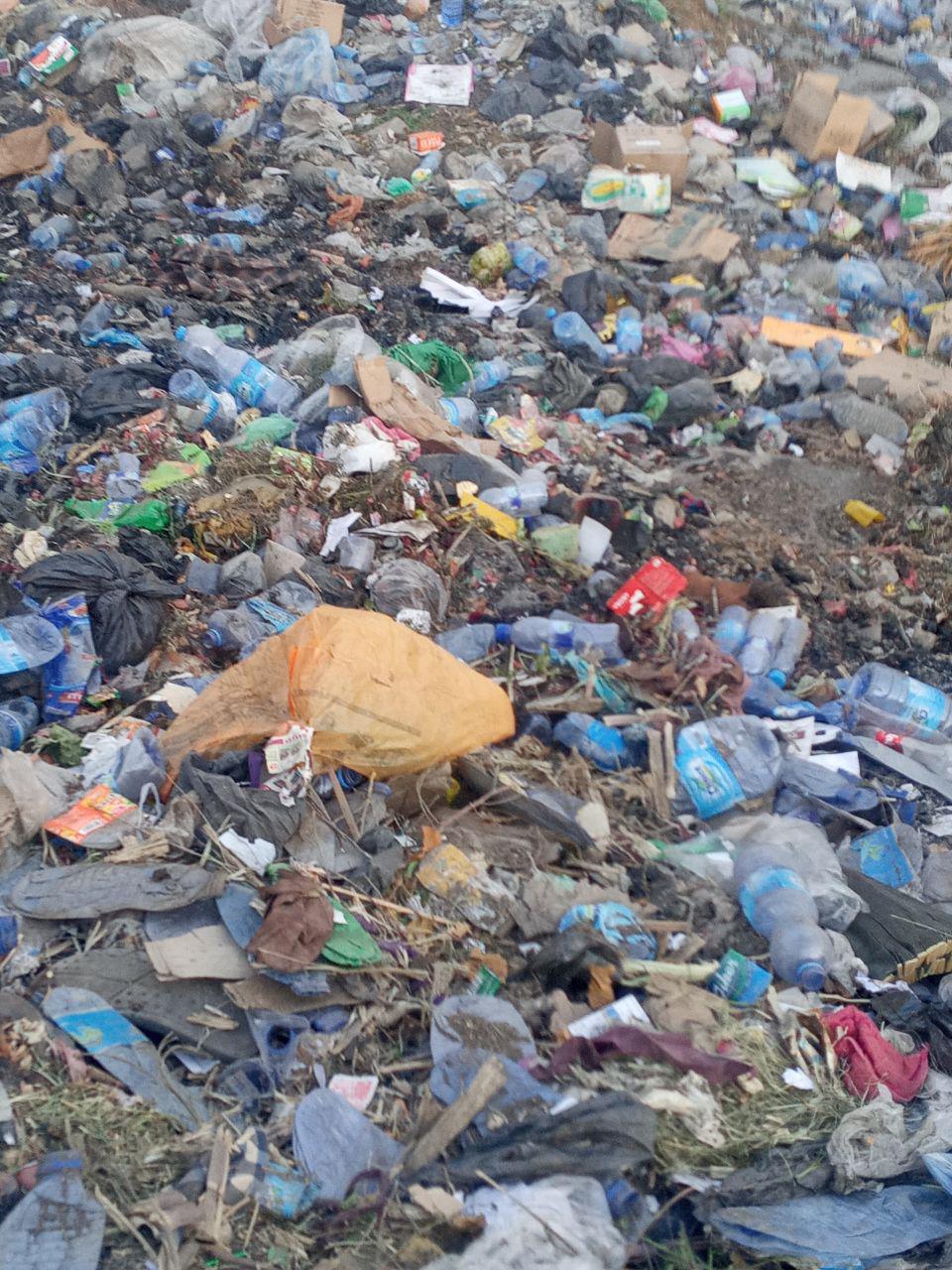

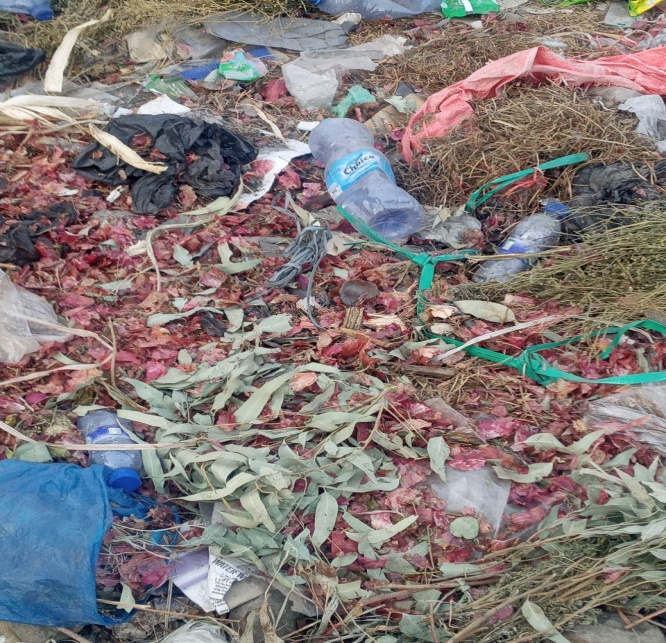


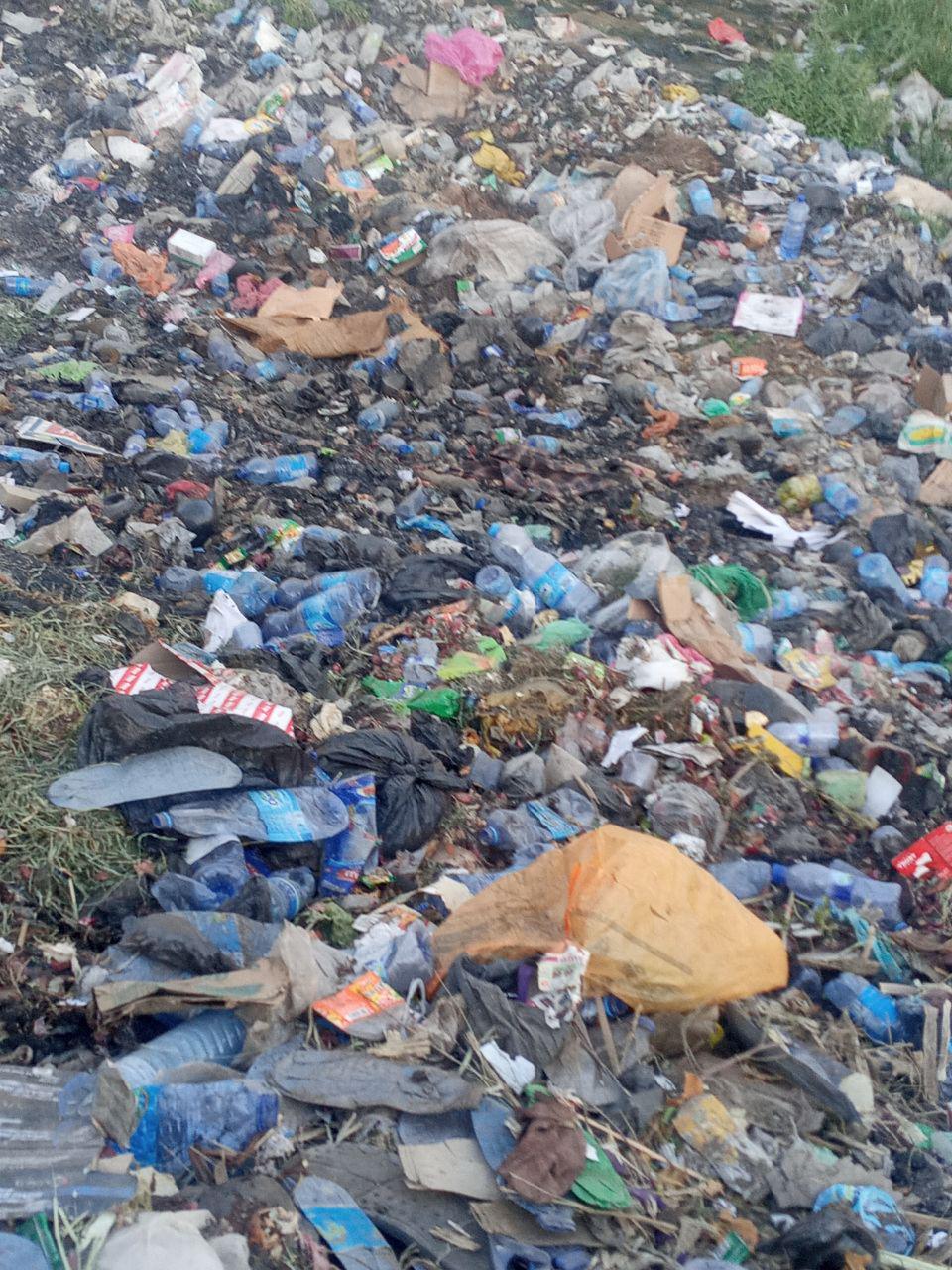

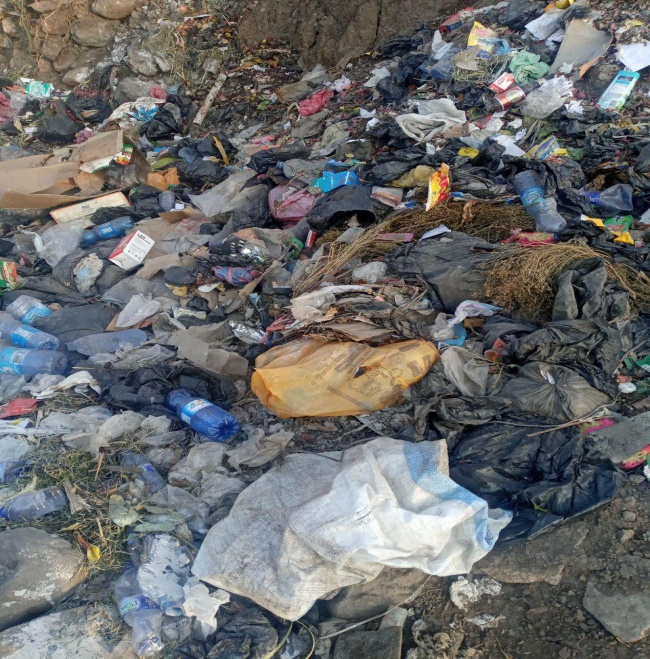


Photo credit: Silamlak Birhanu Abegaz 2019
